# Supplementary material for: Are carbon emissions trading and green financial instruments synergistic? -Comprehensive quantitative research based on content analysis
Source: PLoS One. 2024 Mar 7;19(3):e0298601. doi: 10.1371/journal.pone.0298601 (PMC10919601; doi:10.1371/journal.pone.0298601)
Supplement: S1 Data — (ZIP) [file pone.0298601.s001.zip › Data/地级市Z_F工作报告.zip!/╔╜╢1⁄2/╚╒╒╒╩╨/└┤╫╘╣1⁄2╓┌║┼í╛┬φ┐╦╩2╛▌═°í┐_2013.docx]

各位代表：

　　现在，我代表市人民政府向大会报告政府工作，请予审议，并请市政协委员提出意见。

2012年政府工作回顾

　　刚刚过去的2012年，是我市抢抓机遇、应对挑战、砥砺奋进的一年。面对错综复杂的经济形势和严重自然灾害，市政府紧紧依靠省委、省政府和市委的坚强领导，依靠市人大、市政协的监督支持，依靠全市人民的共同努力，深入贯彻党的十八大和省、市党代会精神，牢牢把握主题主线，立足当前保增长、着眼长远增后劲、加大投入惠民生，全市经济社会保持平稳较快发展，实现了新一届政府工作的良好开局。初步预计，全年实现生产总值1360亿元，比上年增长12%，三次产业比例调整为8.8：54.4：36.8。实现公共财政预算收入78.86亿元、财政支出138.02亿元，分别增长15.1%和11%。完成固定资产投资920亿元，增长20%。实现规模以上工业增加值580亿元、主营业务收入2260亿元，均增长15%。社会消费品零售总额增长15%。完成港口货物吞吐量2.84亿吨，增长11.8%。城镇居民人均可支配收入和农民人均纯收入分别增长14%和15%，居民消费价格涨幅控制在2.5%以内。

　　一、全力做好稳增长调结构工作。一是努力保持工业经济平稳增长。出台了优化发展环境和支持小微型企业的意见，设立了小微企业金融服务中心，对市信用担保公司进行增资。全市信用总量和贷款余额分别比年初增加418亿元和195亿元。扎实推进工业结构调整，壮大优势产业规模。现代威亚发动机三工厂、派沃泰自动变速箱一期、海帝电视、凌云海120万吨精炼糖、石大科技二期、金石石化等项目建成投产，新增汽车发动机产能20万台、变速箱40万台、平板电视30万台。派沃泰二期30万台自动变速箱、亚太森博30万吨液体包装纸和12万吨高档生活用纸、晨曦石化15万吨丁辛醇项目开工建设。全市主营业务收入过10亿元、50亿元、100亿元的企业分别达到46家、12家、4家。二是加快培育现代服务业。完成服务业投资506亿元，增长21%，占固定资产投资的比重达到55%，服务业增加值增长11.5%。深入实施“万村千乡”市场工程和“满意消费惠万家”活动，大力构建农村现代经营服务新体系，成功创建为全国农产品现代流通综合试点城市。加大对现代物流、综合旅游和金融业的扶持力度，日照三运、山东凯达等重点物流项目投入运营，年增加主营业务收入200亿元；全年共接待游客2820万人次，实现旅游总收入182亿元，分别增长15%和20%；华夏银行、浦发银行和3家保险公司在我市设立分支机构，日照沪农商村镇银行开业运营。三是推进现代农业发展。农业生产战胜严重自然灾害，粮食总产达到113.88万吨，创新世纪以来第三个高产年。特色农业面积突破130万亩，新增造林面积13.6万亩，畜牧、水产业稳步发展。日照中心渔港具备开港条件。新增市级以上农业龙头企业56家、农民专业合作社376家。东港区、莒县农产品检测中心建成启用，无公害农产品、绿色食品、有机食品和地理标志农产品达到304个。四是奋力抗击“达维”台风。面对突如其来的强台风袭击，全市上下众志成城，全力做好抢险救灾、灾后重建等工作，深入开展“救灾重建保民生、稳中求进保发展”活动，夺取了抗灾救灾的全面胜利。

　　二、突出抓好重大产业和基础设施项目争引建设。全年开工建设大项目133个，完成投资343.6亿元。尤其令人振奋的是，省委、省政府召开了日照钢铁精品基地开工动员大会，项目即将开工建设。国际海洋城挂牌办公，起步区建设全面展开。日照机场场址获中国民航局审查批准。日照至济南城际铁路完成初步规划。日照港30万吨级原油码头建成投产，30万吨扩建项目获国家核准。日照口岸成为全国首批“世界卫生组织口岸核心能力建设达标单位”。胶州至日照天然气长输管线具备通气条件。山西中南部铁路通道日照段开始铺轨。青临高速莒县段、204国道东港和岚山段、336省道莒县段改建工程竣工通车。220千伏稍坡等输变电工程、户部岭水库向市区调水工程建成启用，龙潭沟水库完成投资过亿元。

　　三、积极推动城乡一体发展。一是着力提升城市规划建设管理水平。10项城建重点工程完成投资21.3亿元，北苗家村、董家滩片区改建全面启动，阳光海岸综合整治山海天南段竣工，完成城中村改造拆迁96万平方米，建设安置楼177.3万平方米。坚持把人本理念融入城市建设，建成城市绿道5条48公里，购置新能源公交车40辆、改建公交候车亭67个，城西供热站投入使用。二是加大农村基础设施投入。完成生态文明乡村建设投资6.3亿元，是历年来投入最多的一年，全市超过50%的村庄配备了环卫一体化设施，新增省级生态乡镇7个。新建改建农村公路1136公里。新增电气化村306个。新建农村综合服务中心119处，农村社区发展到307个。新建改建水利工程1735处，扩大改善灌溉面积25万亩，新增节水灌溉面积10.6万亩。三是全力突破县域经济。制定出台了《关于推动县域科学发展整体提升综合实力的实施意见》，下放审批权限，加大政策资金扶持。通过召开现场办公会的方式，帮助区县解决困难。着眼于带动沿线区域发展，对613和336省道线路进行了调整。在全省县域经济考核中，五莲县、莒县位列跨越发展第一名和第五名，受到省委、省政府表彰。

　　四、坚持开放创新“双轮驱动”。一是积极扩大开放。全年共招引推进总投资5000万元以上的项目524个，实际到位资金297.8亿元；实际到账外资4.2亿美元，增长10%，岚山区成为我市首个实际利用外资突破1亿美元的区县。对外贸易在国际市场持续低迷的情况下实现逆势增长，完成进出口总值249亿美元，增长20%。积极推进与华润集团、山东烟草等大企业合作，中心商务区、城市综合体、滨海新城相继开工建设。主动加强区域经济合作，与青岛市和枣庄、菏泽等鲁南经济带城市签署了合作协议。二是不断深化改革。制定出台了户籍管理制度改革实施意见和配套政策，畅通了进城务工、高校毕业生、外来投资置业人员的落户渠道。着眼于保护北部滨海地区，理顺了山海天旅游度假区管理体制。市级公共资源交易大厅建成启用。完善市区税收属地管理办法，推进部门预算、国库集中支付、投资评审等财税改革，财政管理效益不断提高。深入推进医药卫生体制改革，基本药物制度和基层运行新机制得到巩固完善。我市荣获全国文化体制改革先进地区称号。组建了国际海洋城、滨海新城投资公司和人防资产运营公司，成立了市土地储备直属中心，增强了政府对国有资产和土地资源的运营调控能力。山东蓝色经济区产权交易中心成立，齐鲁股权托管交易中心在我市设立分支机构，4家企业实现挂牌交易，日照港实现再融资31.8亿元。三是努力提高科技创新能力。全国首个国家级蓝色经济引智试验区落户日照。高新区创业服务中心成为国家级科技企业孵化器，新组建院士工作站3家，成立市级以上企业技术中心22家、工程技术研究中心18家。高新技术产业产值增长25%，占规模以上工业总产值的比重提高2个百分点。

　　五、加强民生工作和社会建设。一是加大民生投入力度。全市财政用于改善民生的支出达到87亿元，比上年增长11%，占财政支出的比重为63%，11件为民办实事重点项目全面完成。新增城镇就业5.1万人、农村劳动力转移就业3.3万人，城镇登记失业率控制在2.4%。新农合参合率达到99.99%。城乡居民养老保险参保率达到98.4%，养老保险工作受到国务院表彰。开工建设保障性住房16778套、竣工8675套，分别占年度任务的112%和124%。建设农村住房1.7万户、改造危房7500户。解决了13.8万人的饮水安全问题，农村自来水通村率达到93%。提高了城乡低保、“五保”供养、企业退休人员基本养老金、失业保险金、最低工资和城乡居民基本医疗保险财政补助标准，对617户城乡特困家庭进行结对帮扶，社会福利院、救助管理站建成启用。市中医院通过国家三级甲等中医医院评审。24个援疆项目全部完成年度目标。二是推动教育优先发展。教育投入占财政支出的比例达到21.1%，新建改建公办和公办性质幼儿园140所，完成中小学校舍改造34.5万平方米，农村中小学“两热一暖一改”和图书配备工程深入实施，招录中小学教师600名。中等职业教育免学费扩大到所有农村学生，各级各类学校学生资助政策实现市域全覆盖。高校内涵建设取得新突破，日照技师学院挂牌成立。三是大力加强文化建设。积极创建全国文明城市，城市文明程度指数测评列提名资格城市第7名，新创建省级乡村文明示范区2个。扎实开展“文化建设年”活动，千百重点基层文化工程全面完成，市图书馆开工建设，各级公益文化单位全部免费开放。电影《日照好人》等4部作品获省“文艺精品工程奖”。首届日照文化产业博览会成功举办。承办了中日韩国际帆船赛等体育赛事。四是全面加强社会建设。认真落实计划生育、环境保护、耕地保护基本国策，全面完成人口责任目标，人口自然增长率控制在3.43‰；耕地总量保持动态平衡；城市环境综合整治定量考核保持全省第一，通过了国家环保模范城市复核验收，荣获“2012中国最具生态竞争力城市”称号。服务、管理、执法三位一体的城管机制不断加强，城乡社区创新工作稳步推进。省级双拥模范城(县)实现全覆盖。完善应急管理机制，抓好安全生产和食品药品安全监管。圆满完成了十八大安保等重大任务，社会保持和谐稳定。妇女儿童、老年人、残疾人、红十字、外事侨务、对台事务、民族宗教、打击走私、司法仲裁、国防动员、国家安全、统计、人防、史志、档案、气象等工作取得新成绩。

　　六、努力打造务实高效政府。扎实开展“行政程序年”活动，制定出台并严格执行《关于建立健全依法科学民主决策机制的实施意见》、《市政府工作规则》和《关于进一步加强政府自身建设的意见》，政府决策更加科学民主，行政权力运行更加规范透明。坚持开短会、讲短话，文风会风有了新转变。坚持抓落实、办实事，对决策的事情一抓到底，工作节奏和效率有了新提高。加强政务服务制度创新、流程再造和绩效管理，修改和废止涉及行政强制的规范性文件8件，对38个行政执法机关的自由裁量权予以量化规范，发展环境有了新改善。自觉接受人大的法律监督和政协的民主监督，认真做好人大代表建议、政协委员提案办理工作，办复率、满意和基本满意率均达到100%。扎实做好市长公开电话办理、行风在线等工作，保持了民意诉求渠道畅通。认真贯彻执行《审计法》，审计监督力度进一步加大。严格落实党风廉政建设责任制，扎实推进惩防体系建设，加大行政监察力度，从严查处违纪违法案件，廉政建设取得新成效。

　　各位代表！

　　过去一年，在国内外经济环境复杂严峻的情况下，我市经济社会发展取得这样的成绩来之不易。这是省委、省政府和市委坚强领导的结果，是市人大、市政协和社会各界监督支持的结果，是全市上下攻坚克难、团结奋斗的结果。在此，我代表市人民政府，向辛勤工作在各条战线上的全市人民，向各级人大代表、政协委员，向各民主党派、工商联、无党派人士、各人民团体，向离退休老领导、老同志，向驻日照人民解放军和武警官兵，向所有关心支持日照建设与发展的社会各界人士和海内外朋友，表示衷心的感谢并致以崇高的敬意！

　　当前我市经济社会发展仍然面临不少困难和问题：总体实力不强，结构不优，科技创新能力较弱，转方式调结构的任务艰巨；经济企稳向好的基础不够稳固，保增长的压力较大；城镇化水平不高，县域经济薄弱，城乡发展不平衡的问题较为突出；城乡就业、社会保障、医疗、教育、生态环境、安全生产等工作与群众期盼还有差距；政府部门的办事效率、服务质量有待进一步提高。对此，我们一定高度重视，采取更加扎实有效的措施，努力加以解决。

2013年政府工作任务

　　今年是全面贯彻落实党的十八大精神的开局之年，是实施“十二五”规划承前启后的关键一年，是为率先全面建成小康社会奠定坚实基础的重要一年。市委根据党的十八大提出的新要求，立足日照发展的新阶段，顺应人民群众的新期待，做出了到2017年实现“两个翻番”，率先全面建成小康社会的决策部署。这一奋斗目标令人鼓舞，催人奋进。我们必须着眼长远、立足当前，一步一个脚印，把宏伟蓝图变为现实。

　　根据党的十八大和中央、省、市经济工作会议的部署，今年全市经济社会发展的总体要求是：以邓小平理论、“三个代表”重要思想、科学发展观为指导，深入贯彻党的十八大和省、市党代会精神，紧紧围绕“一个率先、五个日照”的目标，牢牢把握主题主线，坚持“六个更加注重”，以提高增长质量和效益为中心，用好“蓝、红、金”国家战略机遇，稳中求进、好中求快，开拓创新、扎实开局，努力推动经济社会率先创新优质发展。主要预期目标是：生产总值增长11.5%，公共财政预算收入增长13%，固定资产投资增长20%，社会消费品零售总额增长15%，进出口总值增长8%，规模以上工业增加值增长15%，港口货物吞吐量增长9%，城镇居民人均可支配收入增长12%，农民人均纯收入增长13%。居民消费价格涨幅控制在3.5%以内，人口自然增长率控制在4.3‰以内。

　　一、推动经济持续健康发展。一是坚持大项目带动、投资拉动。围绕实施“双十·一城”工程，集中抓好200个大项目，确保年内完成投资340亿元以上。优化投资结构，积极引导资金投向高新技术、现代服务业、农业农村、生态环境和民生等重点领域。全面落实鼓励引导民间投资的有关政策，激发民间资本活力。二是优化企业发展环境。大力推行企业“宁静日”制度，加强政银企合作，努力破解要素制约，为企业松绑加油。实施小微企业成长示范工程，加强融资担保体系和公共服务平台建设，助推中小企业健康成长。三是努力保持外贸增长。积极组织企业参加境内外重要展会，巩固传统市场，拓展新兴市场。下大气力抓好水产品、蔬菜、塑料、五金等外贸转型升级基地建设，带动出口品牌创建。大力发展服务贸易和服务外包，加快外贸转型升级。进一步完善航运中心功能，提高通关效率。四是全力拓展消费市场。深入实施“万村千乡”市场和“农超对接”工程，推动便利消费进社区、便民服务进家庭。积极拓展信息、文化、家政、医疗、健身等新兴消费领域。继续抓好房地产市场调控，增加普通商品房供给，促进房地产业稳定健康发展。认真落实鼓励消费的政策措施，提高居民即期消费水平。

　　二、举力办好几件事关全局和长远发展的大事。(1)把日照钢铁精品基地建设作为“头号工程”，围绕项目开工建设，全力做好保障服务工作，搬迁安置区力争年底前交付使用。精心做好钢铁配套产业园区规划，突出高端加工制造业，打造完整产业链条。(2)开工建设日照机场，力争2015年建成通航。(3)加快日照至济南城际铁路、青日连铁路、岚临和潍日高速公路前期工作，力争早日开工建设。(4)开工建设机场高速、613省道奎山至黄墩段、222省道日照段和胶新铁路电气化改造工程。(5)加快推进奎山客运交通枢纽和城市轨道交通规划建设。(6)加快山西中南部铁路通道、204国道海洋城段和市区段、日兰高速龙山互通立交等在建工程建设。(7)积极推进日照港石臼港区规划调整工作，实施30万吨级原油码头扩建、30万吨级矿石码头等10项港口重点工程。(8)提升保税物流中心建设水平，积极争取设立综合保税区。(9)开工建设“沭水东调”和巨峰水库向岚山供水工程，加速构筑现代水网。(10)新建220千伏石井等13项电力基建工程，提升电力保障能力。

　　三、大力推进产业结构转型升级。围绕“五个转变、八个调整、六个创新”，加快构筑具有日照特色的现代产业体系。一是加快临港产业集群培育。全力推进威亚发动机四工厂、派沃泰变速箱二期、森博浆纸三期、五征工程机械制造、岚桥石化二期、金石石化二期、晨曦石化汽油加氢、金禾博源高纯度柠檬酸等龙头项目，强化“产业链延伸”和“价值链再造”，加快培育钢铁、石化、汽车及装备制造、浆纸印刷包装、粮油食品加工等千亿级、五百亿级临港产业集群，年内确保完成百项重点工业项目投资130亿元。二是推动现代服务业提速增效。加快推进临港物流园、煤炭物流园、钢铁物流园、空港物流园建设，着力推动港口、铁路、机场与经济园区、城镇联动发展。积极发展矿石、煤炭、钢材、粮油等大宗商品交易，抓好凌云海糖业蓝海交易中心和食糖现货交易市场建设，加快打造区域性物流中心。推进尚城花园、杏石温泉等特色旅游项目建设，改造提升五莲山、竹洞天、龙门崮、河山等现有景区。新建一条串联沿海主要景点的滨海旅游绿道，规划一条串联五莲山、九仙山、卧龙山和北部滨海新区的山海观光绿道，将景区景点“串珠成链”。举办中国(日照)文化旅游商品设计大赛。积极培育楼宇经济、总部经济、会展经济和研发设计、软件等生产性服务业，加快发展购物、餐饮、休闲、游乐等生活性服务业，力争年内服务业占GDP的比重提高1个百分点以上。三是加快培育战略性新兴产业。大力实施高新技术产业和战略性新兴产业“双百项目”，扎实推进光电、电子信息、工程装备、新材料、新能源汽车等战略性新兴产业园区规划建设，抓好众山生物、华轩新能源等项目，确保高新技术产业产值增长18%以上。

　　四、加快新型城镇化步伐。把城镇化放在战略全局的高度，突出布局优化、产业支撑、社会保障、户籍改革等重点，推动城镇化提质加速。一是拓展优化城镇化空间布局。按照组团式、链条式发展的思路，以市区、滨海新城、海洋城、岚山城区及莒县、五莲县城为节点，统筹推进城乡规划、基础设施、公共服务一体化，推动城市发展空间向北跨越、向南拓展、向县域延伸，着力打造各具功能特色的经济区和环境优美的宜居区。二是实施一批城建重点工程。(1)开工建设中心商务区淄博路地上交通、地下空间设施和钢铁大厦、资润商务大厦、鲁闽大厦等综合性商务办公设施，完成村居安置楼主体及配套工程建设。(2)实施石臼老城区道路交通、绿化、亮化、学校等设施改造提升工程。(3)新建4条城市绿道。(4)新增100辆新能源公交车，建设3处公交停车场，新建改造100个公交候车亭。(5)抓好艳阳路、济宁路等7条城市道路建设改造。(6)启动东关北路商业街建设和阳光海岸整治续建工程。三是统筹推进城镇建设。加大岚山城区建设投入，提升载体功能。推动莒县城区跨沭河发展和五莲县城整体提升。加快海洋城起步区基础设施建设。高标准推进滨海新城建设。抓好涛雒、城阳两个全国发展改革试点镇，夏庄、街头两个省级示范镇和4个市级示范镇建设，着力打造工业强镇、沿海重镇、现代农业大镇和乡村旅游名镇。按照大社区的理念，积极稳妥地推进城中村连片改造和新型农村社区建设，提升开发档次，让老百姓享受更好的公共服务。全面落实户籍制度改革实施意见，放宽进城落户条件，让城市人气旺起来。

　　五、奋力突破县域经济。一是着力优化县域经济发展环境。用好革命老区扶持政策，建立对口帮扶机制。加大简政放权力度，凡能下放的市级审批权限全部下放到县级。贯通山海西路南湖至莒县城区段，启动潮石路改造项目，推进市县、县域之间基础设施对接，打造新的县域经济发展轴。二是加大产业培育力度。引导各区县立足自身特色和优势，进一步提升园区建设水平，主动对接港口优势和临港产业，积极引进临港工业配套型、产业协作型、农产品加工带动型、财税增收型大项目，不断壮大县域实力。三是加快发展现代农业。深入实施农业十大产业振兴规划和稳定百万吨粮食生产能力建设规划，开展粮食高产创建活动，加大科技兴农力度，积极发展精致高效农业，确保粮食产量稳定在100万吨以上，特色农业突破140万亩。加强现代渔业园区和渔港经济区建设，建成5处海洋牧场。发展现代畜牧业，抓好标准化养殖场建设。全面落实强农惠农富农政策，大力培育种养大户、家庭农场、农民专业合作社、产业化龙头企业、农业社会化服务组织，提高农业生产规模化、标准化、产业化、机械化和品牌化水平。四是继续改善农村基础设施。完成龙潭沟水库主体工程，年内具备蓄水条件。实施4座中型水库和18座小(二)型水库除险加固工程，新建600处小微型水源和5处重点河道治理及拦蓄水工程，建成20万亩旱能浇、涝能排的高标准农田。实施农村饮水安全和山丘区找水打井工程，解决5万人饮水困难、12万人饮水安全问题。基本完成水利工程确权划界。新建改建县乡村道路710公里。新建191个高标准电气化村。

　　六、深入推进开放创新。一是积极扩大对外合作。加大对重点产业、重点区域、重点客商的招商力度，深化与韩国现代、新加坡金鹰、香港华润、山东烟草等大集团的合作，努力实现以商招商的新突破。积极参与中日韩自由贸易区地方经济合作示范区建设。充分发挥开发区的先行先试作用，加快建设开放创新先导区、蓝色产业集聚区、宜居宜业新城区。加大“走出去”力度，依托骨干企业开展境外工程承包和资源开发。积极开展区域合作，扎实做好援疆工作。二是深化重点领域改革。按照上级部署，扎实做好营业税改征增值税试点工作。以鲁南临海产业区综合配套改革和蓝色经济区发展改革试点为主线，加大行政管理体制、土地、财税、金融等领域的改革力度。继续深化行政审批制度改革，精简审批事项。组建日照广播影视集团。积极稳妥地推进事业单位分类改革。大力开展股权托管、股权质押融资业务，完善多层次资本市场体系。全力推动企业上市，年内争取新上市企业2家、挂牌交易企业10家。组建农村商业银行。三是提升科技创新能力。把招商引资与招才引智有机结合起来，进一步加强产学研合作，吸引更多的高等院校、科研院所来我市建立研发中心和产业化基地。充分发挥蓝色经济引智试验区功能，引进一批急需紧缺人才。加强创新基地、科技孵化器、技术中心、创新联盟等平台建设，加快构建以企业为主体、市场为导向、产学研相结合的技术创新体系。大力推进工业化与信息化深度融合，积极发展物联网产业，加快打造“智慧日照”。

　　七、全面加强“生态日照”建设。牢固树立尊重自然、顺应自然、保护自然的理念，着力打造自然、生态、宜居城市。一是切实加强生态环境保护。对水源、山体、森林、湿地及海岸线实行最严格的强制性保护，全面实施大气污染防治、水环境治理、破损山体生态恢复、造林绿化等工程，留住原生态，彰显自然美，让我们的家园永远美丽。二是深入推进生态文明乡村建设。大力实施村庄“硬化、净化、亮化、绿化、美化”工程，加快农村小型污水处理设施建设，推进农村环境连片整治。基本配齐村一级环卫设备，初步建立起“户集、村收、镇运、县处理”的农村垃圾收集处理体系。在市区以村居为单位建设环卫管理用房、公厕和垃圾收集站“三位一体”环卫设施。三是坚定不移地抓好节能减排工作。突出电力、钢铁、水泥、石化、造纸等重点行业，加快淘汰落后产能，扎实推进烟气脱硫脱硝工程，加大既有居住建筑节能改造力度，确保完成年度节能减排目标。四是大力推进资源集约节约和循环利用。严把环保、土地、安全等关口，决不以牺牲环境、资源和安全为代价换取一时的发展。落实最严格的耕地保护和水资源管理制度，大力推进闲置、低效利用土地盘活挖潜，加大中水回用力度。扎实推进再生资源回收利用、垃圾发电等项目建设，促进生产、流通、消费过程的减量化、再利用、资源化。

　　八、高度重视以民生为重点的社会建设。按照“守住底线、突出重点、完善制度、引导舆论”的要求，进一步改善和加强民生工作，扎实办好2013年度为民办实事重点项目。

　　一是实施城乡居民收入倍增计划。(1)健全完善工资正常增长机制，拓宽城乡居民增收渠道，努力实现居民收入增长与经济增长同步、劳动报酬增长与劳动生产率提高同步。(2)年内对4000人进行创业培训，发放1亿元小额担保贷款，扶持1万名城乡劳动者创业就业。(3)年内新增城镇就业2.6万人、农村劳动力转移就业2万人，城镇登记失业率控制在3.5%以内。

　　二是提升城乡社会保障和救助水平。(1)开工建设1万套保障性住房，改造5000户农村危房，新增1000户廉租住房补贴；新增住房公积金缴存职工9000人，为1500户购房家庭发放住房公积金贷款3亿元。(2)进一步提高城乡低保和“五保”供养水平，结对帮扶城乡特困家庭600户，对特殊困难家庭给予临时救助和大学新生入学救助。(3)为395户农村贫困残疾人家庭实施危房改造，对7224名低保重度残疾人每人每月发放生活补贴50元，对300名残疾人提供就业创业扶持。(4)实施城乡困难职工“连心关爱工程”，对2600户特困职工进行救助。(5)建立城市社区居家养老政府购买服务机制，年内在40%的城市社区和20%的农村社区设立老年人日间照料中心，新增养老床位2500张。(6)年内力争完成市殡仪馆迁建和救灾物资储备中心建设。

　　三是改善城乡办学条件。(1)完成学前教育三年行动计划，改扩建100处公办及公办性质幼儿园，鼓励社会力量举办幼儿园。(2)完成29所农村中小学“两热一暖一改”和30所农村中小学食堂建设改造任务。(3)大力推进中小学校舍安全工程和标准化建设，年内改造中小学校舍20万平方米。(4)按照省定标准新建改建2所普通高中。(5)创建3所省级规范化中等职业学校。

　　四是提升医疗卫生保障水平。(1)深入推进医药卫生体制改革，巩固基本药物制度，支持五莲县搞好公立医院综合改革试点。(2)把城乡居民医保补助标准提高到280元，启动新农合大病保险工作，开展城乡困难群众重特大疾病医疗救助试点。(3)为0—6周岁适龄儿童开展一类疫苗免费接种。(4)为500名贫困白内障患者实施免费复明手术。

　　五是全面发展社会事业。深入开展社会主义核心价值体系学习教育，扎实推进全国文明城市创建活动，全面提高人民群众的思想道德素质和城市文明水平。巩固提升“文化建设年”成果，抓好市图书馆和基层公共文化设施建设，扩大城乡“一刻钟”公共文化服务圈。认真做好第十届中国艺术节筹办工作。开工建设丁肇中科技馆。启动新一轮《日照市志》修编。抓好创意产业园和服务外包基地建设，发展壮大文化创意、动漫游戏等文化产业，进一步提升黑陶、绿茶、农民画等文化品牌。做好文化遗产保护工作。加强文化市场管理，净化社会文化环境。广泛开展全民健身活动，办好2013年激光雷迪尔级帆船世界锦标赛。认真落实人口目标管理责任制和计划生育利益导向政策，提高出生人口素质。组织开展第三次全国经济普查。做好国防动员、双拥共建、优抚安置和人民防空工作，积极发展慈善、防震减灾等事业。

六是推动社会管理创新。完善体制机制，注重源头治理，不断提高社会管理科学化水平。深入推进平安日照建设，完善全方位、全天候、全员化的维稳体系，健全社会矛盾纠纷排查和调处机制，强化社会治安综合治理，进一步提高群众的安全感。扎实推进普法和依法治市，开展相对集中行政复议权和仲裁工作。深入开展“和谐城管”创建活动，提升城市管理水平。加强城乡社区建设，创新社会组织管理，依法管理宗教事务，严厉打击走私贩私。严格落实安全生产责任制，加强应急管理机制建设，强化食品药品安全监管，努力保持社会和谐稳定。

　　九、大力加强政府机关作风建设。深入学习贯彻党的十八大精神，严格执行中央八项规定，扎实开展为民务实清廉群众路线教育实践活动。

　　一是坚持执政为民。把人民群众对美好生活的向往作为奋斗目标，把城乡居民收入能不能提高、生活能不能改善、困难能不能解决作为衡量工作成效的根本标准，让人民群众共享改革发展成果。深入基层调查研究，了解真实情况，凡是涉及群众利益的决策都要充分听取群众意见，凡是损害群众利益的做法都要坚决禁止。正确处理执法与服务的关系，进一步提高服务意识和办事效率，坚决查处吃拿卡要和不作为、乱作为、慢作为等破坏发展环境的行为。

　　二是坚持求真务实。树立正确的政绩观，多做打基础、利长远、前人栽树、后人乘凉的工作，创造经得起实践、历史和人民检验的业绩。坚决克服形式主义和官僚主义，进一步改进文风会风，可开可不开的会议一律不开，可发可不发的文件、简报一律不发；除市委、市政府统一安排外，市政府领导成员不参加剪彩、奠基等活动，不发贺信、贺电，不题词、题字；下基层要讲求实效，轻车简从，减少陪同，简化接待。

　　三是坚持依法行政。落实重大决策专家论证、公众参与、合法性审查、风险评估等制度，实行重大决策公开、执行情况评估和责任追究制度。严格依照法定权限和程序行使行政权力，依法保障人民群众的知情权、参与权和监督权。各级政府要主动接受人大的法律监督和政协的民主监督，依法执行人大及其常委会的决议决定，认真做好人大代表建议和政协委员提案办理工作，及时听取各民主党派、工商联、无党派人士、各人民团体和人民群众的意见。

　　四是坚持廉洁从政。带头执行廉洁从政各项规定，严格管好家人、亲属和身边工作人员，清清白白做人，干干净净做事。认真履行党风廉政建设责任制，管好班子，带好队伍。狠刹奢靡之风，从严控制和压缩因公出国、公车购置、公务接待等经费支出。坚持用制度管权、按制度办事、靠制度管人，扎实推进惩防体系建设，支持监察、审计部门依法行使监督权，从严查处违纪违法案件，营造干部清正、政府清廉、政治清明的环境。

　　各位代表！

　　党的十八大为我们指明了前进方向，我市率先全面建成小康社会的宏伟蓝图已经绘就。让我们紧密团结在以习近平同志为总书记的党中央周围，高举中国特色社会主义伟大旗帜，在中共日照市委的坚强领导下，解放思想，埋头苦干，凝聚力量，攻坚克难，为加快建设海洋特色新兴城市，率先全面建成小康社会，谱写全市人民美好生活新篇章而努力奋斗！
